# ﻿Redescription of the Neotropical genus *Agathomerus* (Coleoptera, Megalopodidae, Megalopodinae) and description of three new species from Mexico

**DOI:** 10.3897/zookeys.1227.118934

**Published:** 2025-02-11

**Authors:** Geovanni M. Rodríguez-Mirón, Sara López-Pérez

**Affiliations:** 1 Colección Coleopterológica, Museo de Zoología, Facultad de Estudios Superiores Zaragoza, UNAM, Av. Batalla del 5 de mayo s/n, Col. Ejército de Oriente 09230, Mexico City, Mexico Facultad de Estudios Superiores Zaragoza, UNAM Mexico City Mexico

**Keywords:** Distribution, genitalia, Megalopodina, Megalopodini, new synonyms, spermatheca

## Abstract

*Agathomerus* Lacordaire and *A.pulcher* Lacordaire are redescribed, and three new species of the genus are described: *Agathomerusmagdalenae***sp. nov.**, *A.espinosaorganistai***sp. nov.**, and *A.santiagoi***sp. nov.** Illustrations of the habitus, aedeagus and spermatheca of the four species are provided. *Agathomerusbichito* Monrós, *A.batesi* Baly, and *A.superbus* Pic are proposed as new synonyms of *Agathomeruspulcher* Lacordaire.

## ﻿Introduction

Three genera and 33 species of Megalopodidae are recorded from Mexico (Rodríguez-Mirón 2018; Ordóñez-Reséndiz and López-Pérez 2021); before this work, the genus *Agathomerus* Lacordaire, 1845 comprised 55 species (Appendix 1), ten of which are recorded in Mexico (Rodríguez-Mirón 2018; Rodríguez‐Mirón et al. 2021). *Agathomerus* is one of the most diverse genera of Megalopodidae and is distributed from Mexico to Argentina (Rodríguez-Mirón 2018), which follows the typical Neotropical distribution pattern within the Mexican Transition Zone (Halffter 1964, 2003; Halffter and Morrone 2017; Rodríguez-Mirón et al. 2021).

*Agathomerus* contains the species previously described in *Megalopus* Fabricius that lack costate elytra and a medial conical tubercle on the metaventrite. Monrós (1947) recognized a high degree of polymorphism among the antennae in the genus; he also mentioned that certain species contained in *Agathomerus* should be in different genera. Monrós (1947) established five subgenera (*Mesagathomerus* Monrós, *Agathomeroides* Monrós, *Eugathomerus* Monrós and *Trichagathomerus* Monrós) which comprise the species distributed in Argentina. These subgenera were formed mainly based on antennal shape and length, pronotal shape, and density, type and distribution of pubescence. Pic (1947) created the subgenus Longathomerus Pic, but without indicating diagnostic characters.

Rodríguez-Mirón et al. (2021) performed a phylogenetic analysis of Megalopodidae where *Agathomerus* was recovered as a polyphyletic group. *Agathomerusbivittatus* Lacordaire, 1845 was recovered as a sister group of a large part of the tribe Megalopodini. In the same study, *A.fasiatus* (Dalman, 1823) was found to be closely related to *Barticariacyanipes* (Clark, 1866) and was consequently transferred to the genus *Barticaria* Jacoby & Clavareau while some species found in Mexico were shown to be related to *Mastostethus* Lacordaire. The type species, *A.pulcher* Lacordaire, 1845, was nested within a clade formed by species of different subgenera, leading to their synonymization under *Agathomerus* s. str.

This work aims to redefine the genus *Agathomerus* and illustrate its diagnostic characters. Additionally, three new species of *Agathomerus* are described.

## ﻿Material and methods

The specimens examined were obtained on loan from different collections: National Museum of Natural History, Smithsonian Institution, Washington, DC, USA (**NMNH**, Alexander Konstantinov); Muséum national d’Histoire naturelle, Paris, France (**MNHN**, Antoine Mantilleri), Natural History Museum, London, UK (**NHMUK**, Michael Geiser); American Museum of Natural History, New York, USA (**AMNH**, Lee Herman); Essig Museum of Entomology, University of California, Berkeley, CA, USA (**EMEC**, Peter T. Oboyski), Colección Coleopterológica de la Facultad de Estudios Superiores Zaragoza, UNAM, Mexico City, Mexico (**CCFES-Z**, Ma. Magdalena Ordóñez Reséndiz) and Colección Nacional de Insectos Instituto de Biología, Universidad Nacional Autónoma de México, Mexico City, Mexico (**CNIN**, Santiago Zaragoza Caballero).

Terms for anatomy and male genitalia structures follow Ehara (1954); terms for female genitalia follow Suzuki (1988), Matsumura and Suzuki (2008) and Rodríguez-Mirón et al. (2017). Genitalia were dissected and the tissue was macerated with 10% KOH for 10 minutes. The specimens were examined and measured using a Carl Zeiss Stemi 508 stereomicroscope. Images were taken with an AxiocamMRC5 camera attached to a Zeiss Axio Zoom V16 microscope with a Plan NeoFluar Z objective and 1×10.25 FWD 56 eyepiece at the Laboratorio de Microscopía y Fotografía de la Biodiversidad II, Instituto de Biología, UNAM, and by using a Canon EOS M6 Mark camera, Laowa FX 65 mm f/2.8 2x macro lens and a StackShot macro-rail. Multi-focus images were combined with Zerene Stacker software.

## ﻿Results

### ﻿Megalopodidae Latreille, 1802

**Megalopodinae Latreille, 1802**

**Megalopodini Latreille, 1802**

**Megalopodina Latreille, 1802**

#### ﻿Genus Agathomerus

Taxon classificationAnimaliaColeopteraMegalopodidae

Lacordaire, 1845

86286066-79A1-5D6C-B23F-CCC872ADEC98

**Figure 1.**
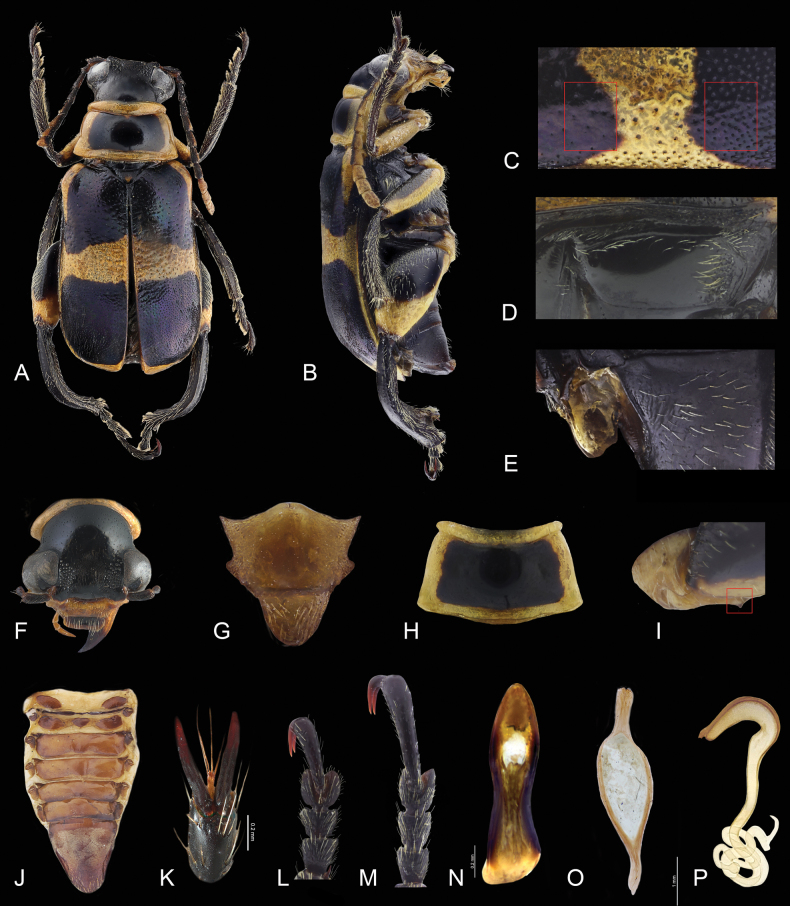
*Agathomeruspulcher***A** dorsal view **B** lateral view **C** elytral punctation **D** metaepisternum **E** mesoventral and metaventral process **F** frontal view **G** mesoscutum **H** pronotum **I** coxa **J** dorsal abdomen view **K** claws **L** protarsomeres **M** metatarsomeres **N** middle lobe **O** tegmen **P** spermatheca.

**Figure 2.**
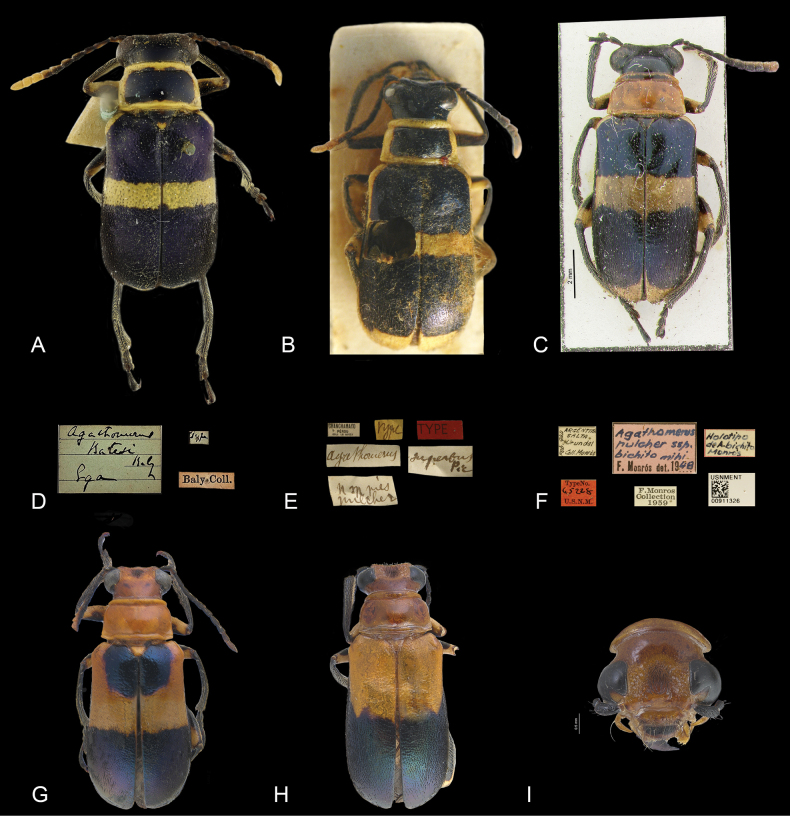
Type specimens and polymorphism among *Agathomeruspulcher***A***A.batesi* Baly, type specimen **B***A.superbus* Pic, type specimen **C***A.bichito* Monrós, type specimen **D** label of type specimen of *A.batesi***E** label of type specimen of *A.superbus* Pic **F** label of type specimen of *A.bichito* Monrós **G***A.pulcher* with elytral disc blue **H***A.pulcher* with elytral disc orange **I** head orange of *A.pulcher*.

**Figure 3.**
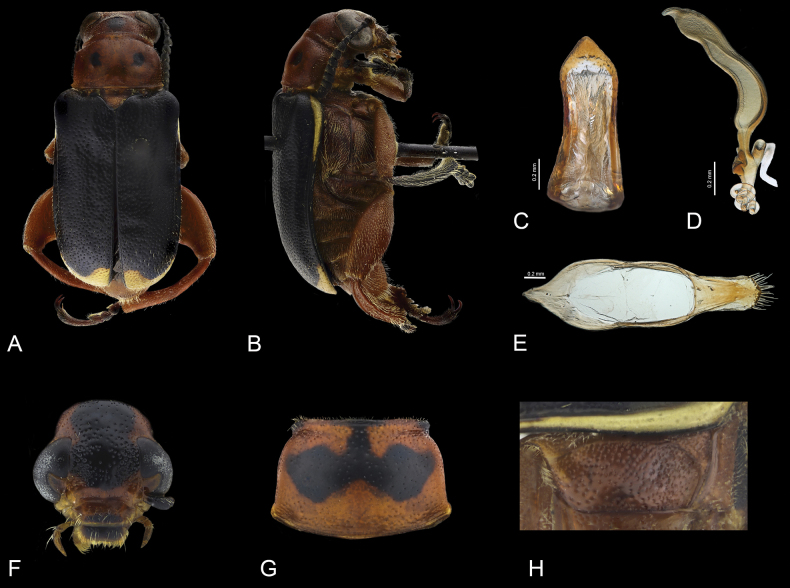
*Agathomerusmagdalenae* sp. nov. **A** dorsal view **B** lateral view **C** middle lobe **D** spermatheca **E** tegmen **F** head in frontal view **G** pronotum **H** metaepisternum.

**Figure 4.**
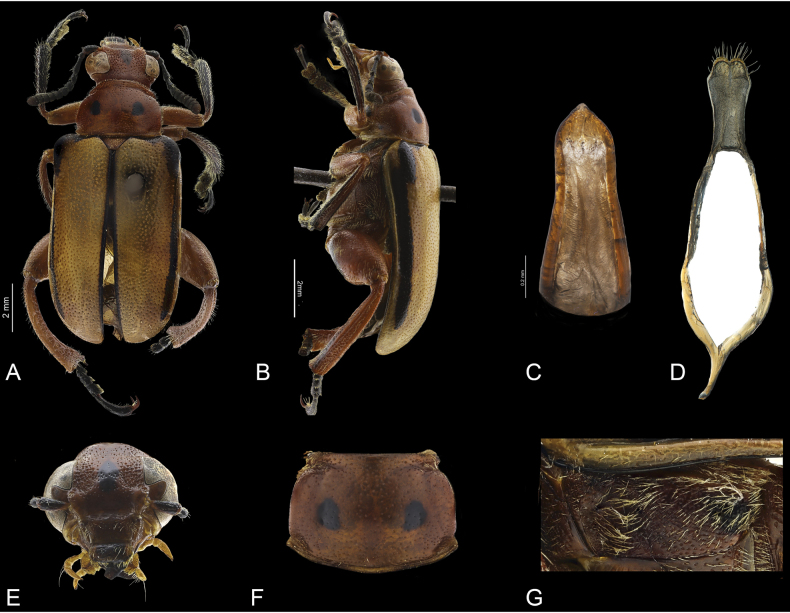
*Agathomerussantiagoi* sp. nov. **A** dorsal view **B** lateral view **C** middle lobe **D** tegmen **E** frontal view **F** pronotum **G** metaepisternum.

**Figure 5.**
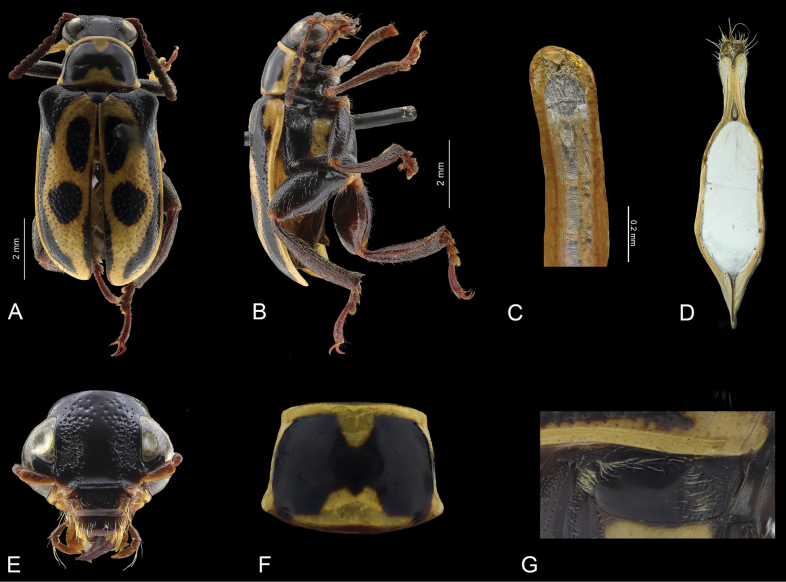
*Agathomerusespinosaorganistai* sp. nov. **A** dorsal view **B** lateral view **C** middle lobe **D** tegmen **E** frontal view **F** pronotum **G** metaepisternum.

Figs 1 , 2 , 3 , 4 , 5

##### Type species.

*Agathomeruspulcher* Lacordaire, 1845, designated by Jacoby and Clavareau (1905).

##### Differential diagnosis.

*Agathomerus* differs from the other genera of Megalopodina by the spatulate shape of the spiculum gastrale; in the other genera it is forked or straight. Moreover, it is also distinguished by the following character states: mesoscutum apex emarginate, metatarsomeres I–III conical, metatarsomeres V longer than I–III and, usually, tergite II with three sclerotized portions. Whereas in *Plesioagathomerus* Monrós, *Mastostethus* and *Homalopterus* Perty the mesoscutum apex is rounded, metatarsomeres I–III are subcylindrical, metatarsomeres V are as long as I–III (except in *Homalopterus* where they are variable); in *Plesioagathomerus* and *Homalopterus* tergite II has two sclerotized portions and in *Mastostethus* tergite II is uniformly sclerotized.

In addition, *Agathomerus* differs from *Mastostethus* by the generally longer antennae and convex metaventrite. In *Mastostethus* the antennae are short and the metaventrite has a conical tubercle medially. It is distinguished from *Homalopterus* by the mandible with a lateral groove, last labial palpomeres fusiform, elytra without costae and long parameres. In *Homalopterus* the mandibles lack a lateral groove, the last labial palpomeres are bullet-shaped, the elytra is costate and the parameres are short. *Agathomerus* can be separated from *Plesioagathomerus* by the abdominal process projected between the metacoxae and by the long empodium. In *Plesioagathomerus*, the abdominal process is short and not projected between metacoxae, and the empodium is short.

*Agathomerus* differs from *Pseudohomalopterus* by the meso- and metaventral processes not joined in lateral view and the emarginate ventral apex of tarsomeres V. In *Pseudohomalopterus*, the meso- and metaventral processes are joined (as in *Mastostethus*), and the ventral apex of tarsomeres V is emarginated and notched. *Agathomerus* is easily distinguished from *Barticaria*, *Bothromegalopus* Monrós, and *Megalopus* by the elongate and robust shape of the body and the subparallel external edges of the elytra. Whereas in *Barticaria*, the body is compact and subcylindrical; in *Megalopus* and *Bothromegalopus*, the elytra are raised behind the scutellum, the elytral suture is depressed, and the body narrows towards the apex (as in *Pseudohomalopterus*).

##### Redescription.

***Head*.** Constricted behind eyes; frontal surface along eye margins with ocellate punctures (Figs 1F, 2I); frons variable, impunctate, glabrous. Interocular space usually transverse. Antennae variable in length and shape, usually reaching anterior edge of elytra (Fig. 1A), with 11 antennomeres; scapes subconical; antennomeres II globose; III shorter than scape; V–X articulated laterally; XI usually ovoid; scape and II–IV with scattered setae; setae on V–XI short and dense. Eyes prominent and notched (Figs 1F, 2I). Frontoclypeal groove conspicuous. Clypeus trapezoidal, punctate; surface of disc with erect pubescence, posterior region with a transverse, translucent band; labrum oblong, surface of disc sparsely pubescent, edge densely so. Mandibles triangular, not toothed; external side with groove, punctate, and with procumbent setae. Maxillary palps with 4 palpomeres, I subcylindrical, II clavate, III shorter than II and IV, IV subconical or bullet-shaped. Ligula bilobed. Labial palps with 3 palpomeres, I subcylindrical and very short, II clavate and longer than III, III fusiform and longer than I. Mentum bilobed. Gula trapezoidal, punctate.

***Thorax*.** Pronotum trapezoidal (Fig. 1H) or bell-shaped (Figs 3G, 4F, 5F), wider than long; base wider than the anterior margin, with anterolateral projections; surface convex, with a groove (either continuous or discontinuous) on the anterior part; posterior part wider than anterior, usually with posterolateral punctures and wide posterolateral projections; posterior edge beaded, as wide as base of elytra. Procoxal cavities closed, procoxae contiguous, conical, protruding; mesocoxae ovate, below level of mesothorax; metacoxae subquadrate, metaxoxal space narrow. Mesoscutum (Fig. 1G) with stridulatory files; apical region rounded or slightly emarginate. Scutellum subtriangular (Fig. 1G). Elytra with external edges parallel (Fig. 1A); surface slightly convex dorsally, punctate; humeri rounded, projecting anterolaterally. Mesanepisternum flat; meso- and metaventral processes not joined (Fig. 1E). Metaepisternum with anterior part concave (Fig. 1D). Leg pairs progressively longer posteriorly; pro- and mesofemur elongate; metafemur robust, elongate, male with or without ventral projections; metatrochanters truncate; tibiae with a row of denticles dorsally; apophysis long and rounded, apex with 2 spurs; pro- and mesotarsomeres I–III subconical (Fig. 1L); metatarsomeres I–III subcylindrical (Fig. 1M); on each leg tarsomeres IV reduced in size; tarsomeres V curved, dorsal edge mucronate, ventral edge emarginate, metatarsomere V longer than I–III combined; claws bifid; empodia long, with more than three setae (Fig. 1K).

***Abdomen*.** Last spiracle smaller than others (Fig. 1J); pygidium subtriangular (Fig. 1J), apically sparsely punctate and sparsely pubescent; sternite I with anterior edge beaded, abdominal process projected anteriorly between metacoxae; in females, last abdominal ventrite concave apically and, on internal surface, short projection into abdominal cavity.

***Male genitalia*.** Aedeagus: dorsal portion of median lobe membranous; struts and lateral margins of median lobe sclerotized (Fig. 1N); struts articulated with edge of aedeagus. Parameres long, fused (Fig. 1O); tegmen ring lanceolate; roof short (Fig. 1O).

***Female genitalia*.** Spermatheca complex (Fig. 1P); proximal part of spermathecal capsule boomerang-shaped, apex with velum; terminal portion of spermathecal capsule elongate. Sternite VIII bifurcate, apex and base trilobate. Spiculum gastrale straight.

#### ﻿ Agathomerus pulcher

Taxon classificationAnimaliaColeopteraMegalopodidae

Lacordaire

74DE65A7-A471-5AD8-8797-67E430CCD308

Figs 1 , 2

Agathomerus pulcher Lacordaire 1845: 675.
Agathomerus batesi Baly, 1859: 199 syn. nov.
Agathomerus superbus Pic, 1916: 20 syn. nov.
Agathomerus bichito Monrós, 1945: 144 syn. nov.
Megalopus injunctus Pic, 1938: 16.

##### Type material examined.

***Holotype*.***Agathomerusbatesi* Baly (designated here) (Fig. 2A, D). ♀, pinned. Original label: “*Agathomerusbatesi* Baly, Lga // type” (NHMUK). ***Syntype*.***Agathomerussuperbus* Pic (designated here) (Fig. 2B, E). Sex?, Glued on cardboard. Original label: “*Agathomerus* // *superfus* pic // TYPE // Type // Chachamayo, Pérou, Coll. Le Moult// illegible” (MNHN). ***Holotype*.***Agathomerusbichito* Monrós (Fig. 2C, F). ♀, Glued on cardboard. Original label: “Argentina, Salta, Urundel, Feb., 944, Coll. Monrós // *Agathomeruspulcher* ssp., bichito mihi, F. Monrós det. 1948 // Holotipo de *A.bichito*, Monrós // Type NO. 65228, U.S.N.M. // F. Monrós Collection, 1959 // USNMENT, QR 00911326” (NMNH).

##### Other material examined.

♂, pinned. Original label: “Perou Chanchamayo, Ch O Schunke // F. Monrós ES Collection, 1959”, (NMNH). ♀. Original label: Pucallpa, Loreto, Peru, Nov. 9, 1946, alt 600 ft (NY). ♂. Original label: “Amazones, Tarapote, M. de Mathan, 4^e^trimestre 1885 // Museum Paris Coll. M Pic”, (MNHN). ♀. Original label: “Amazonas, Brazil, Benjamin Constant, Rio Javary, March 1942 // August Rabaut Collector (AMNH); ♀. *Same data as preceding* “Feb. 15-Mar.15, 1942” (AMNH). ♀, pinned, with genitalia in a separate microvial. Original label: “Peru” // *Agathomeruspulcher* Lac. Det. A.S. Konstantinov, 2014” (NMNH). ♀, pinned. Original label: “Prov. del Sara, Cent. Bolivia, 450 m, 1909, J. Steinbach // F. Monrós Collection, 1959” (NMNH). ♂, pinned, with genitalia in a separate microvial. Original label: “R.A. Salta Pocitos, I852, Coll. A. Martínez”, (NMNH). ♂, pinned. Original label: “Rurreaabaque Rio Beni Bólivia, W M Mann // F. Monrós Collection, 1959” (NMNH). ♀, pinned. Original label: “Peru: Dpt. Cuzco, Prov. Quispicanchis, Quincemil, 6-11-X-1976, Robert Gordon” (NMNH). ♂/♀, pinned, with genitalia in a separate microvial. Original label: “S^1a^. Paulo d Olivenca, M. de Mathan, Juin Juillet 1883” (MNHN). ♂, pinned. Original label: “Pérou, Moyobamba, M. de Mathan, 1888” (MNHN). ♂, pinned, with genitalia in a separate microvial. Original label: “Pérou Tarapoto, Mai à Aout, 1886, M. de Mathan” (MNHN). ♀, pinned. Original label: “Bolivie, Prov. Cochabamba, P. Germain, 1889” (MNHN). ♀. Original label: “Bolij // Jacoby Coll 1909-28a” (NHMUK).

##### Diagnosis.

*Agathomeruspulcher* differs from other species by the following combination of character states: interocular region (Figs 1F, 2I) with a group of fine punctures and erect pubescence; metatrochanter with one tooth (Fig. 1I) ; elytra with two types of punctures (Fig. 1C), anterior region and apex coarsely and sparsely punctate, and posterior region partially and finely punctate.

##### Redescription.

Length 10–12 mm, width 3.8–4.8 mm. Head (Fig. 1F, I) black or yellow; mouthparts yellow; antennae bicolored, antennomeres I–IV black, V–XI black and yellow or last three yellow; pronotum yellowish orange, disc sometimes blue or purple (Figs 1H, 2G). Elytra (Figs 1A, B, 2A–C, G, H) yellow or orange with iridescent blue or purple stripes of variable shape and length, one near base and one subapical. Prosternum yellow, meso- and metathorax black or yellow; coxae generally yellow. Femora mostly yellow, with black maculae dorsally. Abdomen black, with black pubescence.

***Head*** (Figs 1F, 2I). Occiput sparsely punctate or impunctate, interocular region with group of fine points and erect pubescence; ocular canthi surface concave; antennal awning short, not prolonged onto canthi; antennae long (Fig. 1A, B), last four antennomeres surpassing posterior edge of pronotum. Scape as long as antennomere III, II twice as long as III, III 1.2 times longer than V, IV as long as V, V as long as wide, V–X wider than long; XI bullet-shaped; last maxillary palpomeres subconical, apex blunt.

***Thorax*.** Pronotum (Fig. 1H) broader than long, subrectangular, base wider than anterior margin, disc convex, sparsely and shallowly punctate (as on head), anterior portion with clearly defined groove, anterior angles coarsely punctate, lateral margins weakly rounded and glabrous, posterior portion of disc with lateral depressions, posterior margin bisinuate and edged by ridge, posterior lateral angles prominent and with apex rounded. Scutellum (Fig. 1G) flush with elytral surface, coarsely punctate, subtriangular, apex rounded; elytra (Fig. 1A) laterally subparallel, epipleura with rounded edge; humeri rounded, projecting anterolaterally; disc convex, surface with two types of punctures (Fig. 1C), anterior region and apex coarsely and sparsely punctate, and posterior region partially and finely punctate. Metaepisternum (Fig. 1D) with anterior portion coarsely punctate and pubescent, rest impunctate and glabrous; metaventrite convex, shallowly punctate and pubescent; metatrochanter with one tooth (Fig. 1I); pro- and mesofemur slender and elongate, metafemur robust and reniform (Fig. 1B); tibiae curved; metatibiae strongly curved, surface with four faces; dorsal face convex, with longitudinal carina, ventral face glabrous except pubescent on apical region.

***Abdomen*.** Tergite I with two sclerotized regions, tergite II with three sclerotized regions (Fig. 1J); pygidium with apex rounded (Fig. 1J); last abdominal ventrite sparsely punctate, with decumbent pubescence, apex subtruncate. Aedeagus with median lobe (Fig. 1N) strongly sclerotized, narrowing medially, apical region concave, with apex apiculate and deflected. Tegmen (Fig. 1O) slightly sclerotized; parameres with apex pubescent, emarginate and with rounded medial notch; tegmen ring lanceolate, roof slender, 0.8 times shorter than parameres.

**Female.** Femora fusiform, tibiae swollen near apex; last abdominal ventrite with apex emarginate, pygidium with apex rounded. Spermatheca (Fig. 1P) hook-shaped, proximal part of spermathecal capsule with velum short, hook-shaped, terminal portion coiled and notably long, cecum rounded.

##### Distribution.

Argentina, Bolivia, Brazil, Mexico, and Peru (Rodríguez-Mirón 2018).

##### Remarks.

The color pattern in *Agathomeruspulcher* exhibits a high degree of polymorphism (Fig. 2), and because of its wide distribution, specimens from different regions were considered to belong to different species.

After reviewing the specimens from Francisco Monrós’ collection deposited in the NMNH, GRM observed label annotations that Monrós considered *A.bichito* and *A.pulcher* as synonyms. On the other hand, he considered other specimens as different subspecies because of the variation in their coloration. However, these observations were never published.

The type specimen of *Agathomeruspulcher* was collected in Mexico (Lacordaire, 1845), although the exact locality is not specified; for many years this species was considered restricted to this country. However, after the present study, *A.pulcher* is now considered a species with a wide distribution, from Mexico to Argentina. It should be noted that after reviewing main collections from different countries, no specimens of *A.pulcher* from Mexico were found, which calls into question its presence in this country.

#### ﻿ Agathomerus magdalenae sp. nov.

Taxon classificationAnimaliaColeopteraMegalopodidae

D238A924-63A5-5842-8914-B1B9CB5924AD

https://zoobank.org/62DDE802-6E42-4839-B714-C7106D0A0C7B

Fig. 3

##### Type material examined.

***Holotype*.** Mexico • 1♂; Chiapas, Aguacero; 6 June 1987; W. B. Warner leg; pinned, with genitalia in a separate microvial; CNIN. ***Paratypes*.** MEXICO • 1♀; Same data as holotype; CCFEZ-Z. • 1 ♀; Guerrero, Taxco A., Santiago Temixco; 18°28'46"N, 99°37'50.1"W; alt. 1251 m; 23 May 2008; S. López leg.; 15:55; SBC; CCFEZ-Z • 1♂; Oaxaca, Candelaria Loxicha, Portillo del Rayo; 24 May 1990; A. C. Deloya; Bosque tropical y cafetal; CCFEZ-Z.

##### Diagnosis.

*Agathomerusmagdalenae* sp. nov. is similar to *santiagoi* sp. nov. and it is distinguished by the following combination of character states: ocular canthi surface convex, scapes longer than antennomeres III, II twice as long as III, pronotum (Fig. 3G) with a continuous groove on anterior portion, scutellum rounded at apex, metaepisternum (Fig. 3H) with anterior region impunctate and glabrous, rest coarsely punctate and pubescent; metatibiae ventrally flat, parameres (Fig. 3E) with rounded apex, and roof robust. In *A.santiagoi* sp. nov. the ocular canthi surface is concave, scapes are as long as antennomeres III, II half as long as III, pronotum with anterior groove interrupted medially (Fig. 4F), scutellum slightly emarginate at apex, metaepisternum (Fig. 4G) uniformly punctate and pubescent, metatibiae ventrally concave, parameres (Fig. 4D) with a cleft at apex, and slender roof.

##### Description.

Length 10.8–11.6 mm, width 4.7–5.0 mm. Body (Fig. 2A, B) with pubescence yellow; occiput, front, antennae, clypeus, labrum and mandibles black, rest of head, thorax, apical region of femora and abdomen orange; pronotal disc, basal region of femora, tibiae and tarsi black; elytra mostly dark blue, anterior lateral margin and apices yellow.

***Head*** (Fig. 3F). Occiput and interocular region coarsely and sparsely punctate, with long pubescence; ocular canthi surface sparsely convex; antennal awning short, not prolonged onto canthi; antennae barely surpassing posterior edge of pronotum; scapes 1.1 times longer than antennomeres III, II twice as long as III, III 1.5 times longer than V, IV shorter than V, V as long as wide, VI–X wider than long; last maxillary palpomeres subconical, apex blunt.

***Thorax*.** Pronotum bell-shaped (Fig. 3A, G), pronotal disc convex, with two depressions, coarsely and sparsely punctate, (though less coarse than on head), with erect pubescence, anterior groove interrupted medially, anterior angles coarsely punctate, lateral margins rounded and densely pubescent, posterior region with lateral depressions, posterior margin edge with ridge, posterior lateral angles prominent and rounded. Scutellum depressed with respect to elytral surface, subtriangular, emarginate at apex, coarsely punctate; elytra with external margins subparallel, epipleura with rounded edge; humeri rounded, projecting anterolaterally; disc convex, coarsely and sparsely punctate. Metaepisternum (Fig. 3H) mostly coarsely punctate and pubescent, anterior portion impunctate and glabrous; metaventrite convex, coarsely punctate and pubescent; pro- and mesofemur slender and elongate, metafemur robust and fusiform (Fig. 3B); tibiae with pubescence erect, dorsal surface with ocellate punctures and a longitudinal carinae, meso- and metatibiae curved, metatibiae ventrally glabrous, with two incomplete longitudinal carinae.

***Abdomen*.** Tergites evenly sclerotized; pygidium apically sparsely punctate and sparsely pubescent, apex rounded; last abdominal ventrite sparsely punctate and pubescent; setae decumbent; surface concave apically; apical margin subtruncate.

Aedeagus. median lobe (Fig. 3C) narrowing medially, apical region subtriangular, apex rounded. Tegmen (Fig. 3E) sclerotize; parameres setose laterally, apex rounded and with long setae; tegmen ring oblong, roof robust, 1.5 times shorter than parameres.

**Female.** Pronotal disc without depression (Fig. 3G); femora slightly pyriform, tibiae subcylindrical, swollen near apex; meso- and metatibiae slightly curved. Pygidium with apex subtruncate; last abdominal ventrite with subtruncate apex. Spermatheca (Fig. 3D) boomerang-shaped, basal part of spermathecal capsule with velum highly developed. Stem with projection, terminal portion coiled and notably long.

##### Distribution.

Mexico: Chiapas, Guerrero, Oaxaca.

##### Etymology.

The new species is named after Ma. Magdalena Ordóñez Reséndiz, the degree advisor of the authors.

#### ﻿ Agathomerus santiagoi sp. nov.

Taxon classificationAnimaliaColeopteraMegalopodidae

AB6FA32E-DF0A-5FE8-8981-73B35EA2756C

https://zoobank.org/552E61BA-A6C5-4B15-96CC-CEBBDCBA4C6A

Fig. 4

##### Type material examined.

***Holotype*.** Mexico • 1♂; Morelos, 2.5 km, 4 km O Huautla, Estacion CEAMISH; 18°27'671"N, 99°02'475"W; Alt. 940 m, 7–12 July 1996; A. Pérez leg.; ex vegetación; pinned, with genitalia in a separate microvial; CNIN.

##### Diagnosis.

*Agathomerussantiagoi* sp. nov. is similar to *magdalenae* sp. nov. and it is distinguished by the following combination of character states: ocular canthi surface concave, scapes as long as antennomeres III, II half as long as III, pronotum (Fig. 4F) with anterior groove interrupted medially, scutellum slightly emarginate at apex, metaepisternum (Fig. 4G) uniformly punctate and pubescent, metatibiae ventrally concave, parameres (Fig. 4D) emarginated at apex, and slender roof. In *A.magdalenae* sp. nov. the ocular canthi surface is convex, scapes are longer than antennomeres III, II twice as long as III, pronotum (Fig. 3G) with a continuous groove on anterior portion, scutellum rounded at apex, metaepisternum (Fig. 3H) with anterior region impunctate and glabrous, rest coarsely punctate and pubescent; metatibiae ventrally flat, parameres (Fig. 3E) with rounded apex, and robust roof.

##### Description.

Length 11.6 mm, width 4.4 mm. Body (Fig. 4A, B) with pubescence yellow; head, thorax and abdomen orange; mandibles, antennae, protibiae and tarsi black; interocular region and clypeus with a black macula (Fig. 4E); labial and maxillary palpomeres yellow; pronotum with two black maculae (Fig. 4F); elytra yellow with black lateral stripe, elytral suture and humeri black.

***Head*** (Fig. 4E). Occiput coarsely and sparsely punctate, interocular region sparsely punctate except setigerously punctate on midline, setae long; ocular canthi surface concave; antennal awning short, not prolonged onto canthi; antennae barely surpassing posterior edge of pronotum; scapes as long as antennomeres III, II half as long as III, III twice as long as V, IV as long as V, V as long as wide, VI–X wider than long; frontoclypeal groove arcuate; last labial palpomeres bullet-shaped, apex blunt; last maxillary palpomeres subconical, apex blunt.

***Thorax*.** Pronotum (Fig. 4F) bell-shaped, pronotal disc convex, coarsely and sparsely punctate (punctures less coarse and sparser than on occiput), with anterior groove interrupted medially; anterior angles and lateral margins rounded and pubescent, posterior region with lateral depressions, posterior margin edged with a ridge, posterior lateral angles slightly projected; scutellum depressed with respect to elytral surface, subtriangular, apex weakly emarginate, coarsely punctate; elytra with external margins subparallel, epipleura with lateral edge rounded; humeri rounded, projecting anterolaterally; disc convex, coarsely and sparsely punctate. Metaepisternum (Fig. 4G) coarsely punctate and pubescent; metaventrite convex, coarsely punctate and pubescent; pro- and mesofemur elongate, metafemur robust and fusiform; tibiae with pubescence erect, dorsal surface with ocellate punctures and a longitudinal carinae, meso- and metatibiae curved, metatibiae ventrally glabrous with two crenate, longitudinal carinae.

***Abdomen*.** Tergites evenly sclerotized; pygidium sparsely, setigerously punctate, apex rounded; last abdominal ventrite concave, sparsely, setigerously punctate, setae decumbent, apex truncate. Aedeagus: median lobe (Fig. 4C) narrowing towards apex, apical region subtriangular, apex blunt. Tegmen (Fig. 4D) sclerotized; parameres dorsally setose; setae long; apex emarginate; tegmen ring ovoid; roof half as long as parameres.

##### Distribution.

Mexico: Morelos.

##### Etymology.

The new species is named after Santiago Zaragoza Caballero, the PhD advisor of the authors.

#### ﻿ Agathomerus espinosaorganistai sp. nov.

Taxon classificationAnimaliaColeopteraMegalopodidae

F7D7D3B3-9325-5165-995B-FCB74CF5C147

https://zoobank.org/60E626E1-E1C3-4498-AB63-DE1B19C0C5CA

Fig. 5

##### Type material examined.

***Holotype*.** Mexico • 1♂; Sinaloa, El Palmito, 8mi W; 24–64 July // UC Berkeley; EMEC; QR 729245; pinned, with genitalia in separate microvial; EMEC.

##### Diagnosis.

*Agathomerusespinosaorganistai* sp. nov. is similar to *A.signatus* (Klug) and it is distinguished by the following combination of character states: pronotum with anterior groove interrupted medially (Fig. 5F), scutellum with apex subtruncate, male metafemur fusiform and metatibiae curved (Fig. 5B) , and metaepisternum (Fig. 5G) with anterior region coarsely, setigerously punctate, rest impunctate and glabrous. In *A.signatus* the pronotum has a well-defined anterior groove, scutellum apex is rounded, male metafemur are kidney-shaped and strongly curved, and metaepisternum has anterior and posterior regions punctate and pubescent, with rest impunctate and glabrous.

##### Description.

Length 9.6 mm, width 4 mm. Body (Fig. 5A, B) with pubescence yellow; head, thorax and femora black; mouthparts, antennae, tibiae and tarsi brown; pronotum, elytra and metaventrite yellow; pronotal disc with an M-shaped macula (Fig. 5F); elytral humerus black with a black stripe extending near the elytral apex and two spots on each elytron.

***Head*** (Fig. 5E). Occiput sparsely and coarsely punctate, interocular region impunctate and glabrous; ocular canthi surface concave; antennal awning short, not prolonged onto canthi; antennae moderately long, last antennomeres surpassing posterior edge of pronotum; scapes 1.5 times longer than antennomeres III, II twice as long as III, III as long as V, IV shorter than V, V as long as wide, VI–X wider than long; frontoclypeal groove straight; last maxillary palpomeres subconical, apex truncate.

***Thorax*.** Pronotum (Fig. 5F) broader than long, bell-shaped, base wider than anterior margin, pronotal disc convex, sparsely and finely punctate, with short erect pubescence, anterior portion with groove interrupted medially, anterior angles rounded, not projected, lateral margins weakly rounded, posterior margin edged with a ridge, posterior lateral angles weakly projected.

Scutellum depressed with respect to elytral surface, sparsely and coarsely punctate, subtriangular, apex subtruncate; elytra external margins subparallel, epipleura with lateral edge rounded; humeri rounded, projecting anterolaterally; disc convex, sparsely and coarsely punctate. Metaepisternum (Fig. 5G) with anterior portion coarsely punctate and pubescent, posterior portion impunctate and glabrous; metaventrite convex, sparsely punctate; pro- and mesofemur slender and elongate, metafemur swollen; tibiae with pubescence erect, surface convex and with a longitudinal carinae, meso- and metatibiae curved, metatibiae ventrally flat and glabrous with two crenate, longitudinal carinae.

***Abdomen*.** Tergites evenly, weakly sclerotized; pygidium sparsely punctate and pubescent, apex truncate; last abdominal ventrite sparsely punctate, pubescent, setae decumbent, apical region with a transverse groove, apex truncate. Aedeagus: median lobe (Fig. 5C) subparallel medially, apex rounded. Tegmen (Fig. 5D) slender, sclerotized, parameres setose laterally, apex rounded, with long setae; tegmen ring oblong, roof 1.5 times shorter than parameres.

##### Distribution.

Mexico: Sinaloa.

##### Etymology.

The new species is named after David Nahum Espinosa-Organista, the Masters advisor of the first author. The specific epithet is a combination of the last names of Dr David Espinosa-Organista.

## Supplementary Material

XML Treatment for Agathomerus

XML Treatment for Agathomerus pulcher

XML Treatment for Agathomerus magdalenae

XML Treatment for Agathomerus santiagoi

XML Treatment for Agathomerus espinosaorganistai

## ﻿Additional information

### Author ORCIDs

Geovanni M. Rodríguez-Mirón https://orcid.org/0000-0003-0751-4672

Sara López-Pérez https://orcid.org/0000-0002-9925-0186

### Data availability

All of the data that support the findings of this study are available in the main text.

## ﻿Appendix 1

**Table A1.** Checklist of species placed in the genus *Agathomerus* Lacordaire.

**Familia Megalopodidae** Latreille, 1802	
**Subfamily Megalopodinae** Latreille, 1802	
**Tribe Megalopodini** Latreille, 1802	
**Subtribe Megalopodina** Latreille, 1802	
***Agathomerus*** Lacordaire, 1845:673	
Type species: *Agathomeruspulcher* Lacordaeri, 1845, designated by Jacoby and Clavareu (1905).	
***A.flavomaculatus*** (Klug, 1824: 57)	Argentina: Misiones, San Ignacio, Bonpland, Departamento Concepción: Sta. María. Brazil: Rio de Janeiro, San Paulo, Paraná. Paraguay: Puerto Cantera, Sierra de Ambay, Cerro Mirón
*Megalopusflavomaculatus*, Klug, 1824: 57	
***A.almeidai*** Guérin, 1946:205	Brazil: Minas Gerais
***A.affinis*** Jacoby, 1880: 25	Mexico
***A.atripennis*** Jacoby, 1880: 25	Mexico
***A.atripes*** Pic, 1947: 13	
***A.azureipennis*** Lacordaeri, 1845: 680	French Guiana: Cayenne
***A.basalis*** Pic, 1916: 20	Brazil
***A.bifasciatus*** (Klug, 1824: 53)	Brazil: Brazilia
*Megalopusbifasciatus* Klug, 1824: 53	
***A.bipartitus*** Pic, 1955: 13	Bolivia
***A.coeruleus*** Bates, 1866: 81	Brazil: Tapajós
***A.cyaneus*** Clark, 1845: 84	Brazil: Rio grande
***A.cyanopterus*** Lacordaeri, 1845: 677	Brazil
***A.discoideus*** (Klug, 1824: 49)	Argentina. Brazil: Rio de Janeiro, Brazilia
*Megalopusdiscoideus* Klug, 1824: 49	
*Megalopuscinctus* Serville, 1825:319	
***A.dubiosus*** Jacoby, 1876: 808	Mexico
***A.elegans*** (Klug, 1834: 210)	Brazil
*Megalopuselegans* Klug, 1834: 210	
***A.egregius*** (Germar, 1824: 525)	Brazil
*Megalopusegregius* Germar, 1824: 525	
***A.ephippium*** Lacordaeri, 1845: 693	Brazil
***A.espinosaorganistai*** sp. nov.	Mexico: Sinaloa
***A.flavomaculatus*** (Klug, 1824: 57)	Argentina: Misiones, San Ignacio, Bonpland, Departamento Concepción: Sta. María. Brazil: Rio de Janeiro, San paulo, Paraná. Paraguay: Puerto Cantera, Sierra de Ambay, Cerro Mirón
*Megalopusflavomaculatus*, Klug, 1824: 57	
***A.freudi*** Guerin-Meneville, 1852: 437	Mexico
***A.hahneli*** Pic, 1955: 234	“Amazonas”
***A.humeralis*** Serville, 1825: 320	Brazil
*Megalopushumeralis* Serville, 1825: 320	
*Megalopusaxillaris* Klug 1834:212	
***A.hieroglyphicus*** Guérin, 1945:256	Brazil: Paraná
***A.inapicalis*** Pic, 1955: 15	
***A.incomparabilis*** Clark, 1866: 81	Brazil: Espíritu Santo
***A.interrupta*** Pic, 1947: 13	Brazil
***A.lautus*** Bates, 1866: 83	Brazil: Para
***A.latefasciatus*** Pic, 1955: 14	“Amazonas”
***A.lemoulti*** Pic, 1916: 19	Guayana
***A.lineatus*** Guerin-Meneville, 1852: 439	Mexico
***A.luteoreductus*** Pic, 1955: 15	
***A.magdalenae*** sp. nov.	Mexico: Chiapas, Guerrero, Oaxaca,
***A.marginatus*** (Klug, 1824: 51)	Argentina. Brazil. Paraguay
*Megalopusmarginatus* Klug, 1824: 51	
***A.nicki*** Guérin, 1948: 70	Brazil: Sao Paulo, Taipas
***A.nigricollis*** Clark, 1866: 82	Brazil
***A.nobilis*** (Klug, 1834: 210)	Brazil
*Megalopusnobilis* Klug, 1834: 210	
***A.notaticollis*** Clark, 1866: 82	Brazil: Bahía
***A.obliterata*** Pic, 1947: 13	Brazil
***A.pauper*** Bates, 1866: 80	Brazil: San Paulo
***A.pictus*** Lacordaeri, 1845: 691	Brazil
***A.postmaculatus*** Pic, 1955: 15	Mexico
***A.pulcher*** Lacordaeri, 1845: 675	Argentina: Salta (Oran: Urundel). Bolivia. Brazil: Ega. Mexico. Peru.
*Agathomerusbatesi* Baly, 1859: 199 nov. syn.	
*Megalopusinjunctus* Pic, 1938: 16	
*Agathomerussuperbus* Pic, 1916: 20 nov. syn.	
*Agathomerusbichito* Monrós, 1945: 144 nov. syn.	
***A.quadrimaculatus*** (Guérin, 1945: 259)	Argentina. Brazil. Paraguay
*Agathomerusquadrimaculatus* Guérin, 1945: 259	
*Agathomerusvarians* Monrós, 1945: 146	
*Agathomerusquadrimaculatus* Monrós, 1945: 155	
***A.rubrinotatus*** Clark, 1866: 83	Mexico
***A.santiagoi*** sp. nov.	Mexico: Morelos
***A.sallei*** Baly, 1859: 153	Mexico
***A.sellatus*** (Germar, 1824: 524)	Argentina. Brazil
*Megalopussellatus* Germar, 1824: 524	
*Megalopuslimbatus* Mannerheim, 1826:303	
***A.sexmaculatu***s (Kirby, 1818: 444)	Brazil
*Megalopussexmaculatus* Kirby, 1818: 444	
*Megalopusmaculatus* Klug, 1834: 210	
***A.signatus*** (Klug, 1824: 54)	Brazil
*Megalopussignatus* Klug, 1824: 54	
*Megalopuslineatus* Serville, 1825: 320	
*Megalopushenningi* Mannerh, 1826: 302	
***A.simplicipennis*** Jacoby, 1880: 590	Ecuador
***A.succinctus*** Klug, 1834: 212	Brazil
*Megalopussuccinctus* Klug, 1834: 212	
***A.subfasciatus*** (Germar, 1824: 525)	Argentina. Brazil. Paraguay
*Megalopussubfasciatus* Germar, 1824: 525	
***A.testaceus*** (Klug, 1824: 56)	Brazil
Megalopustestaceus Klug, 1824:56	
***A.viduus*** Clark, 1866: 84	Brazil: Rio de Janeiro
***A.violaceofasciatus*** Jacoby, 1888: 64	Brazil. Panama
***A.zikani*** Guérin, 1951: 576	Brazil: Sao Paulo
**Incertae sedis** (Rodríguez-Mirónet al., 2021)	
***A.bivittatus*** Lacordaeri, 1845: 692	Brazil
***A.rufus*** (Klug, 1834: 213)	Mexico
*Megalopusrufus* Klug, 1834: 153	
